# Effectiveness of deep cervical fascial manipulation and yoga postures on pain, function, and oculomotor control in patients with mechanical neck pain: study protocol of a pragmatic, parallel-group, randomized, controlled trial

**DOI:** 10.1186/s13063-021-05533-w

**Published:** 2021-08-28

**Authors:** Prabu Raja G, Shyamasunder Bhat N, César Fernández-de-las-Peñas, Ranganath Gangavelli, Fiddy Davis, Ravi Shankar, Anupama Prabhu

**Affiliations:** 1 Interdisciplinary Centre for Craniofacial and Orofacial pain Research, Department of Exercise and Sports Science, Manipal College of Health Professions, Manipal Academy of Higher Education, Manipal, Karnataka 576 104 India; 2 Department of Orthopedics, Kasturba Medical College, Manipal Academy of Higher Education, Manipal, India; 3 Department of Physical Therapy, Occupational Therapy, Rehabilitation and Physical Medicine, Universidad Rey Juan Carlos, Madrid, Spain; 4 Department of Physiotherapy, Manipal College of Health Professions, Manipal Academy of Higher Education, Manipal, India; 5 Department of Exercise and Sports Sciences, Manipal College of Health Professions, Manipal Academy of Higher Education, Manipal, India; 6 Department of Data Science, Manipal Academy of Higher Education, Manipal, India

**Keywords:** Cervical pain, Musculoskeletal manipulation, Active stretching, Soft tissue therapy, Eye movements, Connective tissue

## Abstract

**Introduction:**

Mechanical neck pain (MNP) is a commonly occurring musculoskeletal condition that is usually managed using electrical modalities, joint mobilization techniques, and therapeutic exercises, but has limited evidence of their efficacy. Pathology (densification) of the deep cervical fascia that occurs due to the increased viscosity of hyaluronic acid (HA) may induce neck pain and associated painful symptoms of the upper quarter region. Fascial manipulation (FM) and yoga poses are considered to reduce the thixotropy of the ground substances of the deep fascia and improve muscle function. The purpose of this study is to investigate the effect of FM and sequential yoga poses (SYP) when compared to the usual care on pain, function, and oculomotor control in MNP.

**Methods:**

This FaCe-Man trial will recruit 160 patients with subacute and chronic mechanical neck pain diagnosed using predefined criteria. Participants will be randomized to either the intervention group or the usual care group, using a random allocation ratio of 1:1. Patients in the intervention group will receive FM (4 sessions in 4 weeks) and SYP (12 weeks) whereas the standard care group will receive cervical mobilization/ thoracic manipulation (4 sessions in 4 weeks) and therapeutic exercises (12 weeks). The primary outcome is the change in the numeric pain rating scale (NPRS). The secondary outcomes include changes in the patient-specific functional scale and oculomotor control, myofascial stiffness, fear-avoidance behavior questionnaire, and elbow extension range of motion during neurodynamics test 1.

**Discussion:**

If found effective, FM along with SYP investigated in this trial can be considered as a treatment strategy in the management of mechanical neck pain. Considering the magnitude of the problem, and the pragmatic and patient-centered approach to be followed, it is worth investigating this trial.

**Trial registration:**

ClinicalTrials.gov CTRI/2020/01/022934. Registered on January 24, 2020 with ctri.nic.in. Clinical Trials Registry – India.

**Supplementary Information:**

The online version contains supplementary material available at 10.1186/s13063-021-05533-w.

## Background

Mechanical neck pain (MNP) is a commonly occurring musculoskeletal condition in the adult population [1]. MNP is defined as non-specific pain in the area of the cervicothoracic junction exacerbated by neck movements [2, 3]. The prevalence of MNP ranges from 5.9 to 38.7% [3]. Almost half of the MNP patients may develop chronic symptoms, contributing significantly to global disability and substantial societal burden [1, 3]. A study that investigated the factors associated with neck and shoulder pain in young adults has reported that increased screen-based activities (prolonged use of computers and mobiles) without any physical activity resulted in a higher prevalence of neck pain among adults [4].

MNP may be associated with temporomandibular pain, oculomotor dysfunctions, headaches, non-otogenic otalgia, and non-odontogenic toothache [5]. Because of the multifactorial presentation of painful symptoms, MNP is often termed as non-specific neck pain and is managed conservatively using electrical modalities, joint mobilization techniques, and therapeutic exercises [3].

A systematic review has shown multiple cervical manipulation sessions may provide better pain relief and functional improvement than medications. The authors have also reported that cervical manipulation and mobilisation present similar results for all outcomes, including pain, function, and patient satisfaction [6]. Considering the association between decreased thoracic mobility and cervical pain as well as the higher risk of cervical manipulation complications, thoracic manipulation is preferred [7]. Also, a review has reported the effectiveness of therapeutic exercises in neck pain patients is uncertain without high-quality evidence [8]. Moreover, the clinical prediction rules pertinent to the conventional management of neck pain in adults are at the preliminary stage which mandates the need for their validation [9].

The mechanosensitive neural tissue is considered a primary feature in cervicobrachial pain syndrome with pain in the upper quarter region (UQR) [10, 11]. Nevertheless, Gangavelli et al. reported that only 19.9% of cases are of neurogenic origin [12]. In addition, Butler had proposed that not all “positive tension tests” indicate adverse neurodynamics, and the neurodynamics tests are not specific enough to indicate abnormal neural mobility [13–15]. In patients with positive neurodynamics tests, there can be diverse problems that are not related to “neural tension” that may create a painful response [14]. A systematic review of anatomical studies has reported that deep cervical fascia (DCF) links the muscles of the UQR, thus forming the in-series myofascial continuity, which may induce nociceptive pain in cervicobrachial pain [16, 17].

**Fig. 1:**
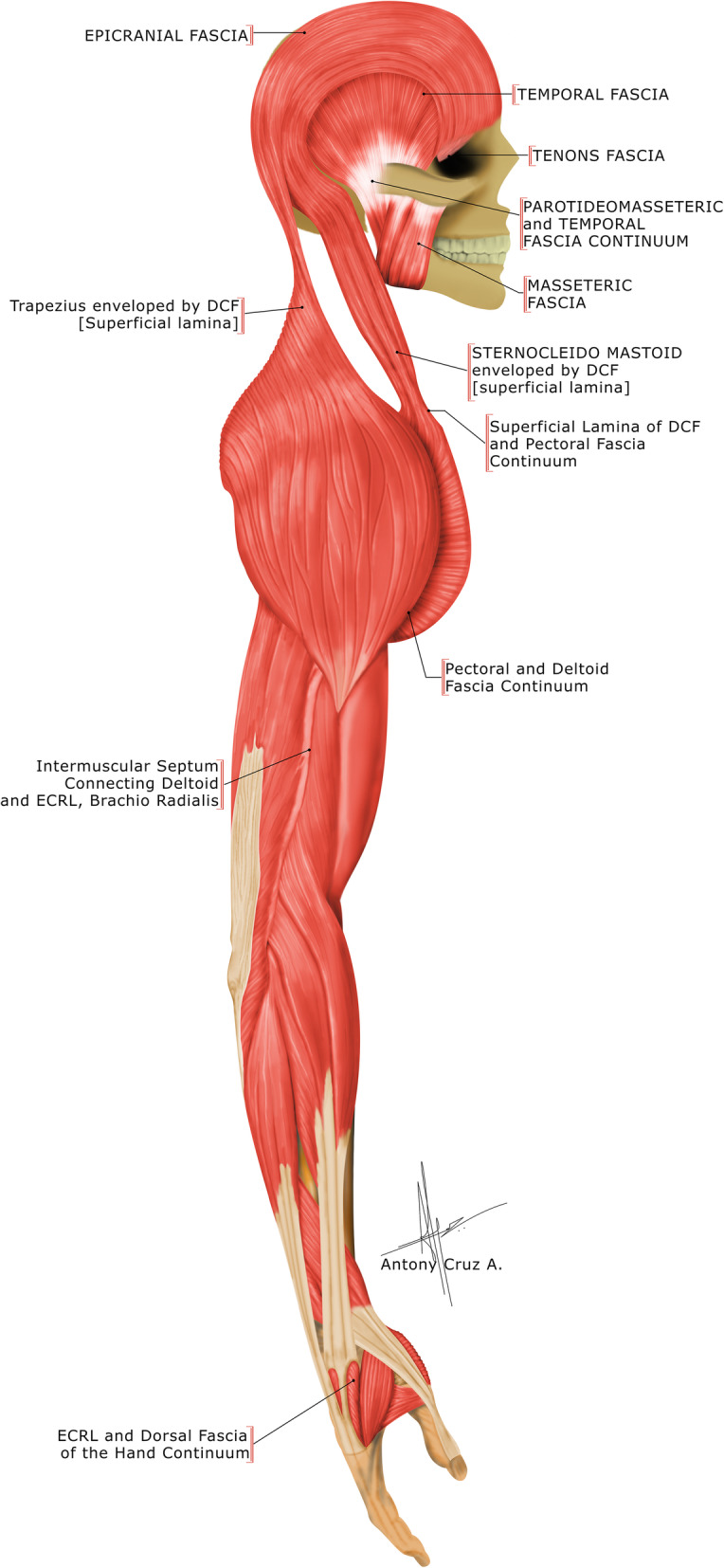
Deep Cervical Fascia and its Continuum (© Antony Cruz & Prabu Raja)

Studies have shown the existence of myofascial expansions, where the DF connects the different muscles of the UQR [18, 19]. The deep cervical fascia (DCF) at the neck has myofascial continuity proximally and distally, forming a myofascial continuum (MC) of the upper quarter. The DCF and its MC that links the head, neck, and upper extremity are illustrated in Fig. 1 [19, 20]. The presence of nociceptors in such connective tissues may convey nociceptive signals directly. Besides, the proprioceptors may change to nociceptors, thus transforming the mechanical stimuli into pain signals. Studies have reported that changes in the hyaluronan of the connective tissues may alter the viscoelasticity of the myofascia resulting in the activation of nociceptors. Thus, dysfunctions of the DCF may be the causative factor in non-specific pain and other symptoms of the eye, head, neck, and arm associated with MNP [17, 21].

Oculomotor dysfunctions associated with MNP are the sensorimotor dysfunctions of the oculomotor system characterized by pursuit, saccade, and convergence deficiencies [22, 23]. EMG studies have revealed an altered activation of the temporomandibular joint and neck muscles in patients with visual defects [23, 24]. Studies have shown an increase in the cervico-ocular reflex (COR) [23] and ocular symptoms such as visual stress, lachrymation in patients with non-specific neck pain attributing it to the impaired receptors at the facets and the deep muscles of the cervical spine [25–27].

The fascia’s inability to extend and accommodate the craniocervical musculature tension is hypothesized to have occurred due to the modification of the DCF in chronic MNP. Restoring the physiological elasticity of the DCF by FM may result in appropriate afferent inputs from the receptors present in the neck muscles that elicit COR, leading to improved oculomotor control.

The increased viscosity of hyaluronan leads to the formation of adhesions and the generation of tensional forces. The adhesions alter the activation of mechanoreceptors, leads to the non-physiologic movement of the joints, resulting in pain and dysfunctions [28–31].

Fascial manipulation (FM) is hypothesized to restore the restricted movement of collagen and elastin fibers within the ground substance. The manipulation of the densified center of coordination (CC) and center of fusion (CF) points might improve the flexibility of the myofascial structures, thereby improving the fascial mobility and the associated symptoms [17, 28, 31]. Besides FM, sequential yoga poses (SYP) also focus on the myofascial lines. It restores fluid flow, decreasing the thixotropy in the ground substances, leading to the muscles’ effective and efficient functioning [32–34].

Patient-reported outcome measures (PROM) such as the numeric pain rating scale (NPRS) and patient-specific functional scale (PSFS) are used in assessing pain and function, respectively [35].

The presentation of various symptoms of the upper quarter with MNP leads to an extensive treatment without any significant treatment effect. Concomitant occurrence of symptoms such as neck pain, headache, and visual dysfunctions may occur due to the impairment in the anatomical fascial connections between the deep cervical fascia (DCF), epicranial fascia, and the tenon’s fascia. A well-designed pragmatic study incorporating manipulation of the DCF, and its continuum in MNP is needed. Thus, the FaCe-Man trial aims to study the efficacy of FM of DCF and SYP when compared to usual care (cervical mobilization (CM), thoracic manipulation (TM), cervicothoracic manipulation (CTM), and home-based therapeutic exercises (TE) on pain, function, and oculomotor control in patients with sub-acute and chronic MNP.

### Objectives

#### Primary research question

Are fascia directed treatment approaches, including 4 sessions of FM in 4 weeks and 3 months of home-based SYP (5 days/week) better than usual care, including 4 sessions of CM, TM, CTM, and 3 months of home-based TE (5 days/week) for improving pain, function, and oculomotor control in patients with subacute and chronic mechanical neck pain?

#### Primary objective

The primary objective of this study is to determine the effectiveness of fascia directed approach that includes FM (4 sessions in 4 weeks) and 3 months of home-based SYP (5 days/week) as compared to 4 sessions of CM, TM, CTM, and 3 months of home-based TE (5 days/week) on pain using numerical pain rating scale (NPRS).

#### Primary research hypothesis

The hypothesis of this study is there will be a significant improvement in MNP patients who undergo fascia-directed treatment approaches (FM and SYP) in reducing pain.

#### Secondary objectives

To study the effectiveness of fascia directed treatment approaches (FM & SYP), in comparison to usual care on function, oculomotor control, elbow extension range of motion (ROM) during ULNT1, viscoelastic properties (tone, stiffness, elasticity) of myofascia, and patient-reported fear avoidance behavior.

#### Trial design

The FaCe-Man trial is a pragmatic, outcome assessor-blinded, randomized, controlled superiority trial with two parallel groups. Randomization will be implemented as stratified (age-based) block randomization with a 1:1 allocation ratio. The trial report is based on the SPIRIT guidelines and checklist-2013 [36] (Additional file 1) and TIDieR guidelines for intervention description and replication [37, 38] (Additional files 2 and 3).

## Methods

### Methods: participants, interventions, and outcomes

#### Study duration

This study period is planned from October 8, 2019, to October 7, 2023.

#### Study setting

Outpatient clinic - Department of Orthopedics at Kasturba Hospitals (recruitment), Center for Sports Science, Medicine, and Research (Interventions and Outcome Measures) and Outpatient Clinic - Department of Speech and Hearing (Outcome Measures), Manipal Academy of Higher Education, Manipal, Karnataka, India.

#### Study flow

**Fig. 2:**
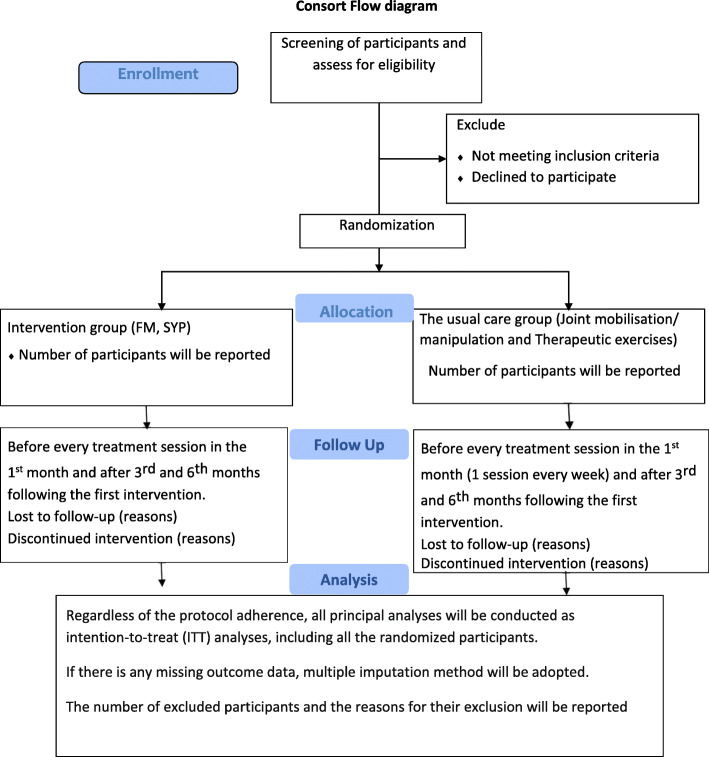
Consort Flow diagram

The study flow is outlined in Fig. 2.

#### Eligibility criteria

##### Inclusion criteria

- Participants: Individuals with sub-acute or chronic MNP for more than 3 weeks.
- Age group: Between 18 and 45 years.

##### Exclusion criteria

Patients diagnosed with any bony lesions, inflammatory diseases, skin infections, vestibular balance disorders, sensory and motor deficits of the UQR, and the history of surgery/trauma to the UQR in the last year or presenting with conditions that are considered as red flags for manual therapy.

### Interventions

In this study, usual care includes therapeutic exercises, cervical mobilization (CM), and thoracic manipulation (TM). Joint mobilization/manipulation will be provided during day 1 and the next three treatment sessions with a 1-week interval between the sessions. Patients in the intervention group will receive FM and instructions regarding home-based SYP. Instructions regarding the home-based therapeutic exercises for the usual care group and sequential yoga poses for the interventional group will be provided during the first treatment session. Regular monitoring of the exercises and SYP will be done during different treatment and follow-up sessions.

#### Usual care group (CM and TM)

Manipulation involves unidirectional thrust movement, whereas mobilization includes smooth oscillatory movements. Large amplitude oscillatory movements and small amplitude at the end range will be used for treating pain and stiffness, respectively. When treating muscle spasm, the rhythm is mainly a sustained position at a point where further movement is restricted by muscle spasm for 1 min in the order of 10–20 s. This sustained progressive position is interspersed with oscillatory movements [39–45].

Therapeutic exercises:

Home-based exercises will be taught, including upper cervical spine mobility and stretching of neck musculature to improve mobility and flexibility. Following this, re-education of the craniocervical flexion (CCF) and progressive training of deep neck flexors and extensors, as well as axio-scapular muscles, will be taught. The details of the usual care are shown in Additional file 2: TIDieR Control group (Usual care group) [37, 38]. The patient’s home-based exercises are illustrated in Additional file 4: Therapeutic exercises - Information leaflet [3].

#### Intervention group (FM and SYP)

The intervention group will receive fascial manipulation and sequential yoga poses.

##### Fascial manipulation

Fascial manipulation (FM) targets the densified points located on the deep muscular fascia termed as the center of coordination (CC) and the center of fusion (CF), which are located on the ligaments, retinacula, and intermuscular septa [17, 19, 28]. The densification may occur at the CC and CF points and are formed due to repetitive abnormal mechanical stresses such as abnormal neck postures. Palpation and movement verifications of the CCs and CFs of the most dysfunctional MFU will be done before selecting the points to be treated for each treatment session. A study on the reliability of movement and palpation assessments in patients with coxarthrosis using the FM method has demonstrated high reliability even if performed by novice FM practitioners [46]. After the identification of a densified center of coordination (CCs) and center of fusion (CFs) by palpation, FM is done by deep friction massage using elbows or knuckles for 5–8 min at each densified CCs and CFs [45, 47, 48]. FM is considered to stimulate these intrafascial mechanoreceptors (paciniform corpuscles, ruffini endings, and interstitial receptors) resulting in altered regulation of motor units thus changing the tissue metabolism and fluid dynamics [19, 48].

##### Sequential yoga poses

Sequential yoga poses focusing on the myofascial continuum of the UQR will be done in the following sequences: triangle pose, extended side angle pose, seated eagle pose, cow face pose, child pose, reverse prayer pose, camel pose, bow pose, and child pose. Each posture will be held for a period of 5 breath cycles, which will be progressed by increasing the number of breathes during these asanas [32–34]. The interventions’ details are given in Additional file 3: TIDieR Intervention group [37, 38]. The patient’s home-based yoga poses are illustrated in Additional file 5: Yoga poses- Information leaflet [32–34].

#### Intervention adherence

Steadfast adherence to exercise-based interventions enhances the effectiveness of the interventions in rehabilitation. Studies have shown that robust exercise adherence may improve neck and back pain patients [49, 50]. The patients will be explained the significance of adhering to study guidelines of home-based rehabilitation exercises and the importance of proper form and exercise execution. The patients will also be advised to contact the investigator on events of any acute flare-up of symptoms.

Reminders via e-mail at regular intervals and Google forms will be sent. Participants will give feedback regarding the intensity, frequency, and duration of their exercise training and the difficulties in performing exercises, improving the adherence and ensuring their participation in the study. These data will be collected once in 2 weeks during the unsupervised exercises following four treatment sessions until the end of 3 months. The investigator will provide leaflets (Additional files 4 and 5) depicting the entire exercise regimen and daily logbook, which will act as reminders if the participants do not have access to the e-mails.

#### Interventions—concomitant care

The concomitant use of NSAIDs, muscle relaxants, and analgesics will be based on the physician’s recommendation and at patients’ discretion. The medications used will be recorded.

### Outcomes

#### Primary outcome measure

- Numeric pain rating scale (NPRS).

##### Numeric pain rating scale

The NPRS is a valid and reliable PROM used for MNP patients. The NPRS has a moderate reliability (ICC = 0.67; [0.27 to 0.84]) [51]. Participants will indicate the intensity of neck pain in the past week. A reduction of 1 point in the NPRS was represented as the minimal clinical important difference (MCID) in patients with chronic musculoskeletal pain [52].

Baseline values will be collected from participants before the first treatment session. Also, the patient’s expected improvement in the NPRS scale following interventions will be assessed. This primary outcome data will be further collected during the 2nd, 3rd, and 4th treatment sessions and the 3rd month and 6th month following the first treatment session. The principal analysis will be performed for changes from baseline to 3- and 6-month follow-up and between the treatment sessions. It will be reported as the difference in the change in the mean between the groups. During analysis, a change score of two in the NPRS scale will be considered as MCID as this score is associated with “much better” improvement [52].

#### Secondary outcome measures

- Patient-specific functional scale (PSFS)
- Oculomotor control tests
  1. Smooth pursuit neck torsion (SPNT) test, saccades using videonystagmography
  2. Near point convergence (NPC) test using RAF ruler
- Fear-avoidance belief questionnaire—physical activity (FABQ-PA)
- Myofascial stiffness using myoton-pro
- Elbow extension ROM during upper limb neurodynamics test 1 (ULNT1) using a goniometer

##### Patient-specific functional scale

The PSFS quantifies the activity limitation in patients where the three most challenging and painful activities will be identified. The total score is the summation of all the activity scores divided by the total number of activities. The minimum detectable change (MDC) for an average rating is 2 points, and the MDC for a single activity score is 3 points [53]. Baseline values will be collected from participants before the first treatment session. Also, the patient’s expected improvement in the PSFS following interventions will be assessed.

The primary analysis will be done for changes from baseline to the last treatment session and 3rd- and 6th-month follow-ups and will be reported as the change in means between the groups.

#### Oculomotor control tests

##### Smooth pursuit neck torsion test

The smooth pursuit neck torsion (SPNT) test assesses the smooth pursuit eye movements with a torsioned neck using videonystagmography (VNG) [54]. In this test, participants will be instructed to follow the target with the eyes without blinking as well as keeping the head still. Initially, smooth pursuit measurement will be performed with the neck and trunk in a neutral position followed by the torsioned position of the trunk to 45° by rotating the trunk on the one side. The same procedure will be repeated, and measurements will be done by rotating the trunk on the opposite side [54, 55]. The SPNT test parameter is the difference between the average gain in the neutral and torsioned positions [25–27, 56].

##### Saccades

Saccades will be measured using VNG, which are rapid voluntary eye movements primarily directed towards stationary targets. In saccades, both eyes move simultaneously between two or more phases of fixation in the same direction. The parameters to be measured in saccades include peak velocity, measured in degrees/second, and latency in milliseconds [25, 26, 54].

##### Near point convergence test

NPC will be measured using an RAF ruler to identify the presence of any convergence insufficiency. Convergence insufficiency (CI) measured by near point convergence (NPC) may be a feature in MNP, thus measuring NPC with neck torsion may differentiate the cervical cause of CI. Giffard et al. have investigated the repeatability and reliability of NPC measurements in neutral and torsion position between the neck pain patients and controls using a Royal Airforce (RAF) ruler. A significant NPC torsion difference was demonstrated in participants with NP compared to controls (*P*=0.01). No significant differences were seen for NPC values in neutral (*P*=0.73). High inter-rater reliability (ICC=0.95) and repeatability (ICC=0.84) was obtained [57].

#### Fear Avoidance Beliefs Questionnaire (FABQ)

The fear-avoidance model (FAM) describes chronic pain development as a psychological process [58]. Although fear-avoidance beliefs (FAB) is considered a key variable in predicting, chronic low back pain (CLBP) FABQ was associated with pain and disability in patients with MNP in increasing order of fear-avoidance beliefs (FAB). FABQ includes a four-item scale, which measures the FAB about physical activity (FABQ-PA), and a seven-item scale, which measures the FAB about work (FABQ-W). The questions of the FABQ refer to “neck pain” instead of low back pain to address the participants with MNP [59]. Higher scores in the FABQ indicate greater levels of fear-avoidance beliefs. Although the association between FAB, pain, and disability in neck pain patients is weaker than in the LBP patients, studies have shown no significant difference in FAB between patients with neck and low back pain. Authors have shown that both FABQ work-related subscales have a substantial relationship with returning to work capability in patients with MNP [59, 60]. FABQ-PA scores include a total score and can be considered as an elevated score when it is 15 or above [58]. Principal analysis will be conducted for changes from baseline to the 3rd- and 6th-month follow-ups and will be reported as the change in the mean between groups. Lee et al. have demonstrated a very good content validity and test-retest reliability of FABQ questionnaire (ICC= 0.81)/(Cronbach’s alpha coefficient= 0.90) [61].

#### Myofascial stiffness

The viscoelastic properties include elasticity (log decrement), stiffness (N/m), and tone (Hz). These mechanical properties will be measured by using Myotonpro on the following muscles: masseter, temporalis, sternocleidomastoid, upper Trapezius, biceps brachii, infraspinatus, and teres minor. Myoton Pro induces oscillation on the skin surface, thus helps in determining the viscoelastic properties of the soft tissues of UQR [62, 63]. The primary analysis will be conducted for changes from baseline to the final treatment sessions and 3rd-month follow-up. The results will be reported as the change in the means between groups.

#### Elbow extension ROM during upper limb neurodynamics test

The mechanosensitivity of the upper limb neural structures may occur due to the nerves’ impaired mobility with their adjacent mechanical interface. The elbow extension ROM (EEROM) is the point in the elbow extension range while performing ULNT1, where the subject experiences pain or discomfort. This degree of elbow flexion will be measured using a standard goniometer [13, 64]. The analysis will be performed for changes from baseline to 3- and 6-month follow-up and between the treatment sessions. The results will be reported as the difference in the change in the mean between the groups.

#### Participant’s timeline

The schedule of enrolment, interventions, and assessments of the participants are outlined in SPIRIT Table 1.

**Table 1:**
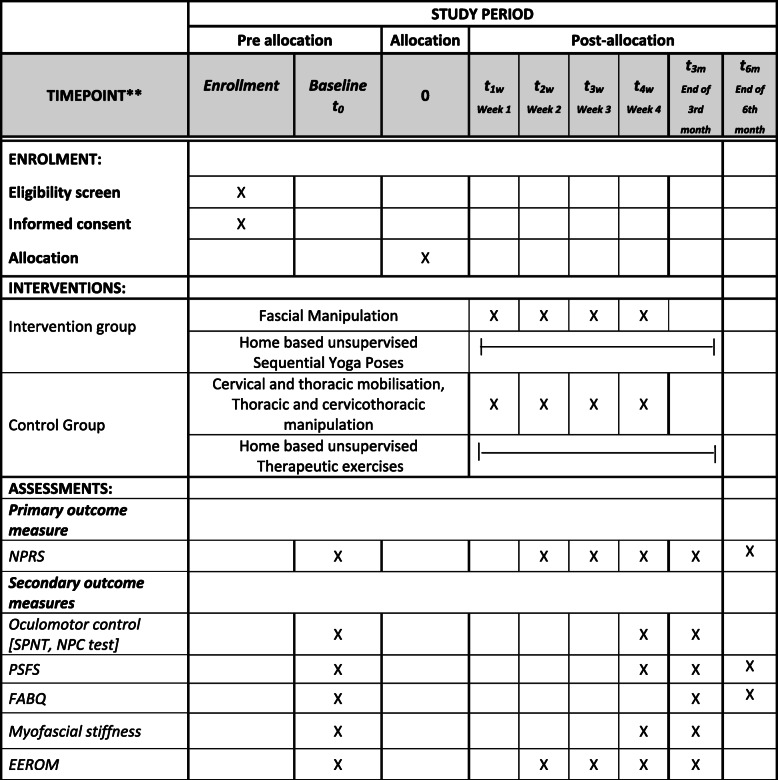
Participants’ timeline. Schedule of enrolment, interventions, and assessments

*Summary of measures to be collected [SPIRIT guidelines 2013]

*NPRS* numerical pain rating scale, *PSFS* patient-specific functional scale, *FABQ* fear avoidance behavior questionnaire, *SPNT* smooth pursuit neck torsion test, *NPC* near point convergence, *EEROM* elbow extension range of motion

#### Sample size

The sample size is estimated based on the primary outcome NPRS. The threshold for MCID in MNP patients with and without UE symptoms is 2.2 and 1.5, respectively [51], whereas the MCID for NPRS in patients with chronic musculoskeletal pain is 1 [52]. Thus, MCID is considered as one in order to have a substantial sample size in this trial.

$$ n=\kern0.5em \frac{2\ \left({Z}_{1-\alpha /2}+{Z}_{1-\beta}\right).\kern0.5em {\sigma}^2\left[\ 1+\left(m-1\right)\rho \right]}{m{d}^2} $$

*d* - MCID = 1

Z_1-α/2_ = 1.96 at *α*=0.05

Z1-β = 1.28 at 90% power

*m* = Number of time points/follow-ups = 5

*ρ* = Intraclass correlation = 0.4

$$ n=\frac{2\times {\left(1.96+1.28\right)}^2\times 2.{5}^2\times 2.2}{5\times {1}^2}=\mathbf{58} $$

Accounting for dropout rate/non-response rate

*N*^*x*^ = *N*/1 – dropout rate; for 10% dropout rate

Required sample size = **64** participants/group.

Total number of participants in the trial = **128**

To detect a difference of one on the NPRS scale (pain intensity) between the groups assuming a standard deviation of 2.5 at a 5% level of significance and 90% power and 10% dropout, the minimum number of participants required in each group is **64**.

#### Recruitment

All participants will be recruited from the Department of Orthopedics, Kasturba Hospital, Manipal. An orthopedician will conduct an initial assessment of the participants with sub-acute and chronic mechanical neck pain. Subjective and objective evaluations including the physical examination will be performed by the orthopedician. In addition, cervical X-rays of the participants with the clinical features of MNP will be done to rule out serious pathology of the cervical spine. The patients with MNP without any red flag symptoms for manual therapy will be recruited. Since the orthopedician is at the first level of recruitment principal investigator will keep him updated regarding the progress of the trial every week to achieve adequate recruitment of the participants.

#### Recruitment status

Anticipated date of enrollment of first participant – July 10, 2020.

### Methods: assignment of interventions

#### Allocation

##### Sequence generation

Random assignment of participants to either control or the intervention group will be conducted at an allocation ratio of 1:1. The sequence will be computer-generated using R software, with two strata, which include group 1: age (18–30) and group 2: age (31–45). Sixteen blocks of 10 people (5 in the control group and 5 in the intervention group) will be used.

##### Allocation concealment mechanism

Neither the investigator nor the outcome assessors will involve in the enrolment or allocation process. The person who will not be involved in the trial will make the randomization schedule and pack the sequentially numbered, opaque, and sealed envelopes, which will be used to randomize the participants.

##### Implementation

The participants who fulfill the inclusion criteria and give consent for participating in the trial will be randomized. The member not involved in the trial will do randomization. Randomization numbers and the therapy group’s corresponding code will be available within the closed envelopes. Staff responsible for recruitment will not receive any information about the allocation of groups.

##### Blinding

The intent of the study, i.e., a comparison between the two different treatment regimens, will be explained to the patients, and the outcome assessor will be blinded.

##### Emergency unblinding

As the interventions can be modified based on the patient’s pain threshold and response, there is less likelihood of circumstances where unblinding will be required. If there is any adverse effect, the participants will be referred back to the orthopedician for further evaluation. The principal investigator after discussing with the orthopedician may modify or discontinue the treatment protocol based on his suggestion.

### Methods: data collection, management, and analysis

#### Data collection methods

##### Data collection

After obtaining written consent, the investigator will assess and evaluate the patients. The same outcome assessors will perform all post-allocation assessments.

##### Retention

The investigator will maintain periodic communication by sending text message reminders for all scheduled treatment as well as follow-up appointments. The investigator will also maintain consistency in implementation and flexible with the study schedule. The participants will be offered to reschedule their appointment within the allotted time interval between the sessions, in case of inability to attend their scheduled treatment session and follow-up assessment.

#### Data management

The patient will fill all the patient-reported questionnaires; the therapist/outcome assessor will fill the outcome measures in paper formats, provided separately. Consequently, all data will be entered electronically, including the Google forms data (online as well as paper-based) will be saved on a secured drive at the study setting. All the original files of the participants will also be stored in a secure and accessible place.

#### Statistical methods

For all the continuous primary and secondary outcomes listed in Table 2, where repeated measurements are to be taken, repeated measures ANOVA will be used. The Student’s *t* test will compare the intervention group (IG) and the control group (CG) during interim assessments. The difference in means will be used for the analysis of continuous variables. All statistical tests will be carried out at a 5% (two-sided) significance level. All analyses will be carried out using the R software, which is open-source software. Patients will be stratified into group 1 ages 18–30 and group 2 ages 31–45. Subgroup analysis will be done based on these groups.

**Table 2:** Summary of reporting outcome measures

Variables/outcomes	Methods of analysis
***Primary outcome measure***	
NPRS (change of pain score during last week) [***∆t***_***0***_ ***→ t***_***3m***_***→ t***_***6m***_ ]	Repeated measures ANOVA
***Secondary outcome measures***	
PSFS (change of functions score) [***∆t***_***0***_ ***- t***_***4***_ ], [***∆t***_***0***_ ***→ t***_***3m***_***→ t***_***6m***_ ]	Repeated measures ANOVA
FABQ [***∆t***_***0***_ ***→ t***_***3m***_ ***→ t***_***6m***_ ]	
EEROM [***∆t***_***0***_ ***→ t***_***3m***_ ]	
Myofascial stiffness [***∆t***_***0***_ ***→ t***_***3m***_ ]	
Oculomotor control [***∆t***_***0***_ ***→ t***_***3m***_ ]	

*NPRS* numerical pain rating scale, *PSFS* patient-specific functional scale, *FABQ* fear-avoidance behavior questionnaire, *EEROM* elbow extension range of motion

***t***_***0***_ baseline, ***t***_***1***_ week 1, ***t***_***2***_ week 2, ***t***_***3***_ week 3, ***t***_***4***_ week 4, ***t***_***3m***_ 3 months following the 1st treatment session, ***t***_***6m***_ 6 months following the 1st treatment session

The mean scores and corresponding 95% CIs will be reported for all the outcomes to be measured between different time points (∆t_0_ → t_6m_) as shown in Table 3. The difference in all outcomes between the interventional and control groups will be reported as shown in Table 4.

**Table 3:** Outcomes (mean, SD)

Outcomes	Baseline/before 1st TS		Before 2nd TS		Before 3rd TS		Before 4th TS		3rd month follow-up		6th month follow-up	
	IG	CG	IG	CG	IG	CG	IG	CG	IG	CG	IG	CG
NPRS	X	X	X	X	X	X	X	X	X	X	X	X
EEROM	X	X	X	X	X	X	X	X	X	X		
PSFS	X	X					X	X	X	X	X	X
Oculomotor functions	X	X					X	X	X	X		
Myofascial stiffness	X	X					X	X	X	X		
FABQ	X	X							X	X	X	X

*TS* treatment session, *IG* intervention group, *CG* control group, *NPRS* numerical pain rating scale, *PSFS* patient-specific functional scale, *FABQ* fear avoidance behavior questionnaire, *EEROM* elbow extension range of motion

**Table 4:** Between-group difference in change of scores

Outcome	Change in scores between the treatment sessions and follow-up														
	***∆t***_***0***_***- t***_***2***_			***∆t***_***2***_***– t***_***3***_			***∆t***_***3***_***- t***_***4***_			***∆t***_***4***_***– t***_***3m***_			***∆t***_***3m***_***– t***_***6m***_		
	IG	CG	Diff (IG & CG)	IG	CG	Diff (IG & CG)	IG	CG	Diff (IG & CG)	IG	CG	Diff (IG & CG)	IG	CG	Diff (IG & CG)
NPRS EEROM															
Outcome measures	Change in scores between treatment sessions and follow-up.														
	***∆t***_***0***_***– t***_***4***_						***∆t***_***0***_***– t***_***3m***_***– t***_***6m***_								
	IG			CG		Diff (IG & CG)	IG			CG			Diff (IG & CG)		
PSFS FABQ															
Outcome measures	Change in scores between treatment sessions and follow - up.														
	***∆t***_***0***_***– t***_***4***_						***∆t***_***4***_***– t***_***3m***_								
	IG			CG		Diff (IG & CG)	IG			CG			Diff (IG & CG)		
Oculomotor control Myofascial stiffness															

*Diff* difference, *IG* intervention group, *CG* control group, *NPRS* numerical pain rating scale, *PSFS* patient-specific functional scale, *FABQ* fear-avoidance behavior questionnaire, *EEROM* elbow extension range of motion

***t***_***0***_ baseline, ***t***_***1***_ week 1, ***t***_***2***_ week 2, ***t***_***3***_ week 3, ***t***_***4***_ week 4, ***t***_***3m***_ 3 months following the 1st treatment session, ***t***_***6m***_ 6 months following the 1st treatment session

#### Statistical methods—analysis population and missing data

Regardless of the protocol adherence, all key analyses will be conducted as intention-to-treat (ITT) analyses, including all randomized participants. If there is any missing outcome datum, multiple imputation methods will be done, so that a complete dataset will be available for the ITT analysis.

### Methods: monitoring

#### Data monitoring

##### Data monitoring committee

No separate Data Monitoring Committee (DMC) will be formed, as the Institutional Research Committee members will perform the roles of DMC. No formal stopping guidelines and corresponding interim analysis are planned.

##### Harms

The primary investigator will record the treatment-related adverse events, including any abnormal signs and acute exacerbations of symptoms during the assessment or treatment sessions. In case of the requirement of medical evaluation, the participants will be referred to the respective medical department.

##### Auditing

The Doctoral Advisory Committee (DAC) members will conduct regular audits.

## Discussion

This FaCe-Man trial will investigate the effectiveness of the fascia-directed treatment approach compared to usual care in patients with MNP. If found useful in improving patient-reported functions and pain, the intervention can be used as an effective strategy in managing MNP and their associated plethora of symptoms. The research question appears to fulfill the FINER criteria, as described in Table 5 [65, 66]. As MNP contributes significantly to global disability and substantial societal burden, the “problem base” is large enough, which needs to be addressed. Also, concerning “the context placement,” the prior evidence shows a lack of knowledge in terms of the DCF continuum and their involvement in causing musculoskeletal pain. It is also essential to study the effectiveness of fascia directed approach addressing the impaired myofascial continuum of the UQR. The FaCe-Man trial may contribute to fill this research gap.

**Table 5:** Appraisal of FaCe-Man trial

Features	FaCe-Man trial
Problem base	Considering the prevalence and chronicity of MNP *, it needs to be addressed.
Context placement	Systematic assessment of prior evidence reveals a research gap
Information gain	Large enough to be informative
Pragmatism	Pragmatic approach to reflect the real life
Patient-centeredness	Patient centric
Value for money	Focuses on larger group of population with MNP
Feasibility	Feasible
Transparency	Method, data, and analyses will be verifiable and unbiased

*MNP Mechanical Neck Pain

Adapted from Ioannidis JPA (2016). Why most clinical research is not useful. PLOS Medicine 13 [6]

This study is designed for multiple follow-ups at different time points, which would improve the quality of the trial. The pragmatic approach will be followed in this trial, thus reflecting the clinical practice situation. Moreover, the patient-reported outcomes such as pain and patient-specific functions, which are the usual symptoms associated with neck pain, would also improve the trial’s patient-centeredness.

Using SPIRIT and TIDieR guidelines will improve the transparency of the study methods and analysis, thus enhancing the relevance of the trial and their results.

Appraisal of the FaCe-Man trial and the FINER Criteria for a good research question is shown in Tables 5 and 6.

**Table 6:** FINER criteria for a good research question

FINER criteria	FaCe-Man trial
**Feasible**	
*Adequate number:*	
Subject	↑ MNP * patients
Expertise	Multi-disciplinary trial
*Affordable*	Yes
*Manageable*	It can be completed within the allotted time
**Interesting**	Investigator/research community/clinicians
**Novel**	Myofascial continuum and force transmission Fascia directed approach
**Ethical**	Low to moderate risk Clearance from Institutional Research and Ethics Committee
**Research**	This study will improve scientific knowledge and clinical practice. Influence future research

*MNP Mechanical Neck Pain

Adapted from Hulley et al. Designing clinical research, 3rd ed.

### Trial status

**Issue date**: October 8, 2019

**Protocol amendment number**: Amendment 01: modified on January 8, 2020 with changes in inclusion age criteria, sample size, and follow-up

**Table:** 

	Initial	(Amendment 01) modifications
Participant’s age group	Age group: 18–65 years	Age group: 18–45 years.
Sample size	Total number of participants= 120 (60 participants in each group)	Total number of participants= 126 (58 participants in each group)
Procedure	3-month follow-up	Included 6-month follow-up following the first intervention

Date of first recruitment: July 08, 2020

Tentative date of final recruitment: August 10, 2022

## Supplementary Information

**Additional file 1.** SPIRIT (Standard Protocol Items: Recommendations for Interventional Trials) checklists (2013).

**Additional file 2 **TIDieR Control group (*Usual care group*).

**Additional file 3.** TIDieR Intervention group.

**Additional file 4.** Therapeutic exercises - Information leaflet.

**Additional file 5.** Yoga poses - Information leaflet.

## Data Availability

Upon publication of the study results, the corresponding author will provide data if the scientific journal requires a full dataset. There are no contractual agreements that limit the dataset access for the investigator.

## Abbreviations

FaCe-Man trial: Fascia cervicalis manipulation trial
MNP: Mechanical neck pain
PAM: Patho-anatomical model
TBC: Treatment-based classification
DF: Deep fascia
UQR: Upper quarter region
EEROM: Elbow extension ROM
COR: Cervico-ocular reflex
DCF: Deep cervical fascia
MC: Myofascial continuum
FM: Fascial manipulation
CC: Centre of coordination
CF: Centre of fusion
IG: Intervention group
UCG: Usual care group
NPRS: Numeric pain rating scale
PROM: Patient-reported outcome measures
SYP: Sequential yoga poses
CM: Cervical mobilization
TM: Thoracic manipulation
PSFS: Patient-specific functional scale
SPNT: Smooth pursuit neck torsion
FABQ: Fear-avoidance belief questionnaire
ULNT1: Upper Limb Neurodynamics Test
