# Supplementary material for: Effectiveness of deep cervical fascial manipulation and yoga postures on pain, function, and oculomotor control in patients with mechanical neck pain: study protocol of a pragmatic, parallel-group, randomized, controlled trial
Source: Trials. 2021 Aug 28;22:574. doi: 10.1186/s13063-021-05533-w (PMC8399821; doi:10.1186/s13063-021-05533-w)
Supplement: Supplementary file 2 — Additional file 2 TIDieR Control group (Usual care group). [file 13063_2021_5533_MOESM2_ESM.docx]

***The TIDieR Usual care description***

**Item 1: Usual care description**

Cervical mobilisation/Thoracic manipulation and Therapeutic exercises, including stretching/strength and conditioning.

**Item 2: Rationale**

The rationale for the interventions is described in the study protocol.

**Item 3: Materials**

The leaflet includes:

A brief description of the aim, content and rationale of the intervention and the specific procedure of the treatment strategies.

Pictures and descriptions of the relevant exercises.

Information on details of progression and regression of exercises.

Details of the advantages, disadvantages and risks related to the intervention.

Contact details of the primary investigator.

**Item 4: Procedures**

Patients in the Mobility / Exercise and conditioning subgroup of the control group will receive cervical spine mobilisation and thoracic spine manipulation by the therapists. Also, instructions will be provided on home-based unsupervised exercises focusing on improving joint mobility as well as the strengthening of the muscles of the cervicothoracic region.

Initially, instructions to the aim and rationale of the interventions will be provided orally with the help of the information leaflet.

The following points are covered in this information:

Bearable pain is OK, as long as it is tolerable. This is not a sign of danger with chronic pain, where the pain system may likely not functioning normally and may not indicate potential damage.

The flare-up of symptoms following exercises is used to guide the need for the regression of exercises. Increased symptoms lasting for more than 24 hours may indicate poor form or high intensity of exercises, which shall be modified according to the instructions provided in the leaflet.

Treatment procedure: Even though cervical manipulation showed better effects than analgesics, NSAIDs, muscle relaxants for improving pain and function, considering the rare chances of inducing adverse effects, cervical mobilisation is preferred over cervical manipulation. Thus only cervical mobilisation and thoracic manipulation will be performed in this study ([1](#_ENREF_1)).

***Cervical mobilisation***

Cervical mobilisation will precede before the manipulation of thoracic spine.

The mobilization will be applied at each cervical spine level as two 1-min repetitions based on the following Maitland’s grades of mobilisation, where the intensity and frequency will vary based on the participant’s condition.

Grades of joint mobilization:

Grade I: A slow, small-amplitude movement that does not take the joint capsule to the limit of available joint motion.

Grade II: A slow, larger amplitude movement that does not take the joint capsule to the limit of available joint motion.

Grade III: A slow, large-amplitude movement that takes the joint up to and slightly through the limit of available joint motion and into tissue resistance.

Grade IV: A slow, small-amplitude movement that is performed through the limit of available joint motion and into tissue resistance.

Rhythm: Smooth oscillatory movements will be performed. When treating pain, the large amplitude of motion will be used based on the symptom response. While treating stiffness and slight pain at the end range technique will be of small amplitude at the limit of their range. When treating muscle spasm, the rhythm is mainly a sustained position at a point where further movement is restricted by muscle spasm for a duration of 1 minute in the order of 10-20 seconds. This sustained progressive position is interspersed with oscillatory movements. If the joint is very painful or presence of protective muscle spasm, manipulation will be avoided ([2](#_ENREF_2)).

Cervical mobilisation for bilateral symptoms:

Postero-anterior central vertebral pressure Postero-anterior U/L vertebral pressure (Both sides) Longitudinal movement

*Posterior-anterior central vertebral pressure:*

Patient position: Prone with the forehead resting on the palm of the patient.

Therapist position: At head end of the couch with pad of the thumbs on spinous process of the spine to be mobilized and the fingers straddle the sides of the neck.

Technique: After lifting the neck slightly into flexion using the fingers, oscillatory movements will be performed using the tips/ pads of the therapist’s thumbs over the spinous processes of participant’s cervical spine.

*Posterior-anterior U/L vertebral pressure:*

Similar to posterior-anterior central vertebral pressure, oscillatory pressure is directed postero- anteriorly over the articular process of the spine. The same procedure can be done at C2 articular process with and without 30° of rotation to differentiate C1/C2 and C2/C3 joints.

*Longitudinal movement:*

Patient position: supine with neck supported by the therapist and in level with the end of the couch. Neck in in midway between flexion and extension.

Therapist position: At the head end of the couch with the patient’s neck cradled firmly by grasping the patients chin with the left hand and with the right hand grasp the occiput region.

Technique: The oscillatory movement longitudinally elongating the patient’s neck.

*Cervical mobilisation for unilateral (U/L) symptoms:*

Postero-anterior U/L vertebral pressure Transverse vertebral pressure

*Transverse vertebral pressure:*

Patient position: Prone with the forehead resting on the palm of the patient.

Therapist position: At the side of the patient's asymptomatic side with the pad of one thumb on the lateral surface of the spinous process and other thumb reinforcing it.

Technique: Laterally directed oscillatory movements will be performed towards the painful side

***Thoracic manipulation:***

*Cervicothoracic and Upper thoracic manipulation (Rotation gliding C7–T3)*

Therapist position**:** At the head end of the client

Patient positioning**:** Patient prone with chin resting on couch and arms hanging over the edge of the couch or against sides of the participants. Initiate side bending opposite to the side to be treated and rotation to the same side of the treatment side.

Palpation of contact point: Left side hypothenar eminence of the therapist will be placed against the transverse process of T3 on the side to be treated and the right hand against the temporal region of the patient’s head.

Procedure for the manipulation:

Rotate the cervical and upper thoracic spine to the side opposite to that of the lateral flexion**.** After achieving an appropriate prethrust tension, the thrust is delivered in the direction of the patient’s axilla along with a minimal and rapid increase of neck rotation ([3](#_ENREF_3)).

*Thoracic Manipulation (Rotation gliding T4–T9):*

Therapist position**:** Standing at the left side of the client.

Patient positioning**:** Patient prone and arms hanging over the edge of the couch or against the side of the trunk.

Procedure for the manipulation: After locating the two opposite side transverse processes of the contiguous vertebra, therapist’s left hypothenar eminence will be placed on the right transverse process of the distal vertebra and the right hypothenar will be placed on the left transverse process of the proximal segment firmly. Then the therapist lean forward, shifting the body weight to the upper limbs and the hypothenar eminences thereby moving the therapist’s centre of gravity forwards. Along this downward pressure, caudad and cephalad directed forces will be delivered through the left and right hypothenar eminences, respectively, which will provide a force on the transverse processes ([3](#_ENREF_3)).

***Therapeutic exercises***

Participants will be taught self-mobilisation of the upper cervical spine and stretching of the neck musculature to improve mobility and flexibility. Following this, re-education of the CCF and progressive activation of the deep cervical flexors and extensors, as well as axio-scapular muscles, will be taught.

*Specific muscle lengthening exercise (Stretching):*

Stretching of suboccipital, levator scapulae, upper trapezius and sternocleidomastoid will be taught. To elongate the muscle tissue, the stretch position should be held for 20–30 seconds, repeated 3–5 times a day.

After re-educating the craniocervical movement, training the deep neck flexors and extensors will be done to improve the recruitment of the deep stabilizing musculature.

*Co-contraction of the neck flexors and extensors:*

Following the patient's ability to recruit the DCF and DCE, co-contraction of the cervical flexors and extensors by performing a self-resisted isometric rotation in supine or sitting will be added. The patient is instructed to perform craniocervical nodding before performing an isometric rotation.

*Retraining scapular control:*

After retraining the scapular orientation, training to improve the endurance of the synergistic muscles of the scapula will be done in prone to achieve scapular control. Progressive retraining of the scapular control can be done using different positions and movement of the arms ([4](#_ENREF_4)).

**Item 5: Providers**

The primary investigator will provide manual therapy techniques and therapeutic exercises. If the principal investigator is unable to complete one or more intervention sessions, he will depute another trained physiotherapist. Therapists will focus on the form of exercises. The therapist will remind the participants about the importance of adherence ([5](#_ENREF_5)).

**Item 6: How**

The instructions of the therapeutic exercises will be delivered face-to-face as well as demonstrated individually to each participant according to their conditions.

**Item 7: Where**

The face-to-face instructions and the demonstrations of the therapeutic exercises, as well as the joint mobilisation/ manipulation procedures, will be provided in a selected intervention room at the study site. After learning the exercises, patients will be advised to adhere to the home-based rehabilitation regimen. Therapeutic exercises will be performed in the study setting during the follow-up session following the joint mobilisation/manipulation performed by the investigator on the participants.

**Item 8: When and How much**

Cervical mobilisation and thoracic manipulation:

Four joint mobilisation/manipulation sessions are planned. The first session is to be held immediately following the baseline assessment of all outcomes. Similar procedures will be performed during 2^nd^, 3^rd^ and 4^th^ follow up sessions with a 1-week interval in between the sessions.

Therapeutic exercises:

Therapeutic exercises have to be performed following all four FM sessions under supervision. The participants will be asked to perform unsupervised exercises at least five days a week from the beginning of the first treatment session until the end of the 3^rd^ month. The progression or regression of these exercises will be based on the instructions in the leaflet.

**Item 9: Tailoring**

Joint mobilisation: Joint mobilisation exerted by the therapist on the participants will vary according to the condition and the tolerance of the participants.

Therapeutic exercise: A standard protocol for progression or regression of therapeutic exercises will be based on pain response. In case of a flare-up of symptoms lasting more than a day, the intensity can be reduced, and the form of these movements can be corrected by looking at the instructions in the leaflet.

**Item 10: Modifications**

Any modifications to the intervention will be reported in the primary trial report.

**Item 11: Adherence**

Lack of adherence to home-based exercise intervention is a key issue while investigating the effect of an intervention. Previous studies have monitored adherence using log-books, which was filled by the patients. These self-reported measures of adherence may have limitations ([6](#_ENREF_6)). Adherence in this study will be improved by sending reminders via e-mail at regular intervals. Also the weekly diary using google forms, where the link will be sent to their mobile phones so that participants can give detailed feedback regarding the intensity, frequency and duration of their exercise training. Along with this, leaflets depicting the entire exercise regimen will be provided, which will act as reminders. Correction and monitoring of the form of the therapeutic exercises will be done at each follow-up visit.

The investigator will give the adherence reminders at the initial intervention and each subsequent intervention sessions. The investigator will also explain the importance of performing the proper execution of all prescribed exercises.

References:

1. Gross, Langevin P, Burnie SJ, Bedard-Brochu MS, Empey B, Dugas E, et al. Manipulation and mobilisation for neck pain contrasted against an inactive control or another active treatment. The Cochrane database of systematic reviews. 2015(9):CD004249.

2. Maitland G HE, Banks K, English K. Maitland’s vertebral manipulation 7th ed: Elsevier 2005.

3. Gibbons P TP. Manipulation of the spine, thorax and pelvis. 4th ed: Elsevier; 2016.

4. Fernández-de-las-Peñas C CJ, Huijibregts PA. Neck and Arm Pain syndromes. Evidence-informed screening, diagnosis and management. Churchill Livingstone,

Elsevier; 2011.

5. Boyling JD JG. Grieves modern manual therapy. The vertebral column 3rd ed: Elsevier; 2004.

6. Bollen JC, Dean SG, Siegert RJ, Howe TE, Goodwin VA. A systematic review of measures of self-reported adherence to unsupervised home-based rehabilitation exercise programs, and their psychometric properties. BMJ open. 2014; 4(6).
