# Supplementary material for: Effectiveness of deep cervical fascial manipulation and yoga postures on pain, function, and oculomotor control in patients with mechanical neck pain: study protocol of a pragmatic, parallel-group, randomized, controlled trial
Source: Trials. 2021 Aug 28;22:574. doi: 10.1186/s13063-021-05533-w (PMC8399821; doi:10.1186/s13063-021-05533-w)
Supplement: Supplementary file 3 — Additional file 3. TIDieR Intervention group. [file 13063_2021_5533_MOESM3_ESM.docx]

***The TIDieR Intervention description***

**Item 1: Intervention description**

Fascial manipulation of the deep cervical fascia and its myofascial continuum/ Sequential yoga poses.

**Item 2: Rationale**

The rationale for the interventions is described in the study protocol.

**Item 3: Materials**

The leaflet includes:

A brief description of the aim, content and rationale of the intervention and the specific procedure of the training strategies.

Pictures and descriptions of the relevant exercises.

Information on details of progression and regression of exercises.

Details of the advantages, disadvantages and risks related to the intervention.

Contact details of the primary investigator.

**Item 4: Procedures**

Patients in the intervention group will receive fascial manipulation treatment by the therapist as well as instructions to perform home-based, unsupervised yoga poses in a sequence. The progression of these poses can be done by increasing the number of breaths in each pose. First, instructions to the aim and rationale of the interventions will be provided orally with the help of the information leaflet.

The following points are covered in this information:

Fascial manipulation may cause mild swelling and slight bearable pain during and after the treatment. The soreness may persist on the treated points, which will subside within 48 hours. The painful symptoms may or may not refer to the adjacent areas. Advice to apply heat on the locations of the different segments where fascial manipulation is performed.

Yoga poses help in improving flexibility.

Bearable pain is OK, as long as it is a tolerable stretch pain. Such symptoms are not a sign of danger with chronic pain, where the pain system is likely not functioning normally and may not indicate potential damage.

The flare-up of symptoms following exercises is used to guide the need for the regression of exercises. This means that flare-up of symptoms lasting for more than 24 hours indicates that the intensity of the training is high, and exercise intensity and frequency should be decreased.

*Treatment procedure:*

*Fascial Manipulation*

Assessment of all the Myofascial Units (MU) along the six myofascial sequences as well as the myofascial spirals of the upper quarter region will be assessed for densification. After the identification of densified and rigid Centre of co-ordination (CCs) and Centre of fusion (CFs) points, the interventions will be given in the form of fascial manipulation on the densified CCs and CFs. This includes deep friction massage using elbows or knuckles or pad of the fingers of the therapists, for a duration of 5-8 minutes. Antagonistic myofascial sequences also will be targeted, and similar assessment/ treatment procedures will be followed (4).

*Sequential Yoga Poses:*

Sequential Yoga Poses (SYP) focusses on the myofascial continuum in the following sequence:

Triangle pose, Extended Side Angle pose, Seated Eagle pose, Cow face pose, Child pose, Reverse Prayer pose, Camel Pose, Bow pose and Child pose. Each posture will be held for a period of 5 breath cycles. This is progressed by increasing the number of breaths in each pose. Each poses to be done in the following order: Right 🡪 Left 🡪 Right 🡪Left ([1](#_ENREF_1)), ([2](#_ENREF_2)).

The participant will receive instructions of the SYP, which is a home-based interventions performed by the participants by themselves, on the first day following FM. Supervised poses have to be performed following all four FM sessions. The form and the progression of these poses will be monitored during their follow up visit for FM. The participants will be asked to perform unsupervised yoga poses at least five days a week from the initial treatment session till the end of 3^rd^ month when the final assessment of all outcomes will be done.

**Item 5: Providers**

The primary investigator, trained in providing the FM interventions as well as in teaching the yoga poses, will deliver the interventions. If the principal investigator is unable to complete one or more intervention sessions he will depute another trained physiotherapist. Training will focus on correcting the form of the different yoga postures as well as reminding the participants about the importance of adherence.

Item 6: How

The primary investigator will provide treatment by manipulating the deep fascia of the upper quarter. The instructions of the SYP intervention will be delivered face-to-face as well as demonstrated individually to each participant.

**Item 7: Where**

The face-to-face instructions will be provided in a selected intervention room at the study site. Exercises will be performed at home

**Item 8: When and How much**

FM: Four intervention sessions are planned. The first session is to be held immediately following the baseline assessment of all outcomes. Manipulation of each densified CC points along the antagonistic sequences will be done for 5-8 minutes (Stecco L, 2004). Similar procedures will be performed on the densified points following the assessment of points of densification in the deep fascia. The same procedure will be done during 2^nd^, 3^rd^ and 4^th^ treatment sessions with an interval of 1 week between the sessions.

SYP: Supervised yoga poses have to be performed following all four FM sessions. The participants will be asked to perform unsupervised yoga poses, at least five days a week, from the initial treatment session until the end of 3^rd^ month following the initial visit. Each yoga pose will be held for a duration of 5 breaths initially according to the participant's ability and then progressed by increasing the number of breaths in the different asanas.

**Item 9: Tailoring**

FM: The pressure or friction exerted by the therapist on the participant will vary according to the pain threshold of the participants. Higher the pain threshold of the participants, the application of friction by the therapist will be higher.

SYP: A standard protocol for progression or regression of yoga poses will be based on the pain response of the participants. In case of flaring up of symptoms, lasting more than a day, or if the participants are finding it challenging to perform the pose, the number of breaths in the hold position can be reduced. In case if a participant can perform the pose comfortably, progression can be made by increasing the number of breaths in the different yoga poses.

Item 10: Modifications

Any modifications to the intervention will be reported in the primary trial report.

**Item 10: Modifications**

Any modifications to the intervention will be reported in the primary trial report.

**Item 11: Adherence**

Lack of adherence to home-based exercise intervention is a key issue while investigating the effect of an intervention. Previous studies have monitored adherence using log-books, which was filled by the patients. These self-reported measures of adherence may have limitations ([3](#_ENREF_3)). Adherence in this study will be improved by sending reminders via e-mail at regular intervals. Also, the weekly diary using google forms, where the link will be sent to their mobile phones so that participants can give detailed feedback regarding the intensity, frequency and duration of their exercise training. Along with this, leaflets depicting the entire exercise regimen will be provided, which will act as reminders. Correction and monitoring of the form of the therapeutic exercises will be done at each follow-up visit.

The investigator will give the adherence reminders at the initial intervention and each subsequent intervention sessions. The investigator will also explain the importance of performing the proper execution of all prescribed yoga poses.

**References**

1. Myers T. Anatomy Trains 2nd ed: Churchill Livingstone, Elsevier 2009.

2. Iyengar BKS. The illustrated light on Yoga: Harper Collins ; 1997.

3. Bollen JC, Dean SG, Siegert RJ, Howe TE, Goodwin VA. A systematic review of measures of self-reported adherence to unsupervised home-based rehabilitation exercise programmes, and their psychometric properties. BMJ open. 2014;4(6).

4. Stecco L, Fascial Manipulation for Musculoskeletal Pain: Piccin; 2004.

5. Schleip R FT, Chaitow L, Huijing P. The Tensional Network of the Human Body: Churchill Livingstone, Elsevier; 2012.
