# Supplementary material for: Effectiveness of deep cervical fascial manipulation and yoga postures on pain, function, and oculomotor control in patients with mechanical neck pain: study protocol of a pragmatic, parallel-group, randomized, controlled trial
Source: Trials. 2021 Aug 28;22:574. doi: 10.1186/s13063-021-05533-w (PMC8399821; doi:10.1186/s13063-021-05533-w)
Supplement: Supplementary file 4 — Additional file 4. Therapeutic exercises - Information leaflet. [file 13063_2021_5533_MOESM4_ESM.docx]

**Therapeutic exercises:**

The aims are 1) To improve the mobility of the upper cervical spine and the flexibility of the muscles of the cervical spine 2) To retrain the craniocervical movement and scapular control

3) To improve the activation of the deep cervical flexors and scapula thoracic stabilizers. Participants will be taught self-mobilisation of the upper cervical spine and stretching of the neck musculature. Following this, re-education of the CCF and progressive activation of the deep cervical flexors and extensors as well as axio-scapular muscles will be taught.

It is OK to feel mild pain during exercises as long as it is tolerable. If the symptoms flare up or persist for more than a day, then reduce the intensity and correct the form of exercises. Resume the usual training routine once the acute exacerbation of symptoms subsides.

For queries: Contact Prabu Raja G, 8867591333, prabu.raja@manipal.edu.

***Self-mobilization of the upper cervical spine:***

CO / C1 flexion: By placing the clasped hands under the skull and stabilizing the neck, perform cranio-cervical nodding followed by lifting the head in a cephalic direction (towards the ceiling) (Fig 1).

Fig 1


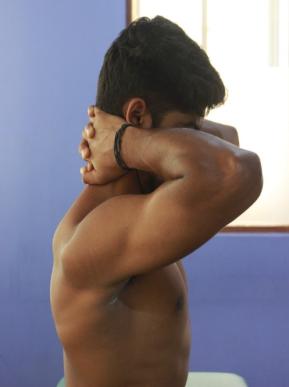

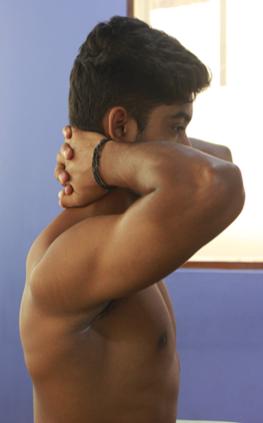


Self mobilisation - CO / C1 flexion (Fo[o (Fig 1

***Specific muscle lengthening exercise (Stretching):***

To elongate tissue, the stretch position should be held for 20–30 seconds, repeated 3-5 times in a day.

*Sub-occipital muscles*: By leaning against the wall and perform cranio-cervical nod. Hold that tucked chin position for 20–30 seconds (Fig 2).

*Cervical extensors:* By maintaining the contact of the thorax against the wall, performing cervical flexion with a tucked chin posture and holding that position, stretches the long cervical extensors

(Fig 3).

*Upper Trapezius:* After keeping the arm behind the back, neck flexion (bending forwards), side flexion (bending sideways) away from the side to be stretched and rotation to the same side to be stretched along with the depression of the scapula (Fig 4).

*Levator scapulae*: After keeping the arm behind the back, neck flexion (bending forwards) followed by side flexion (bending sideways) and rotation away from the side to be stretched along with the depression of the scapula (Fig 5).

*Sternocleidomastoid*: The muscle can be stretched by side flexion away and rotation towards the side to be stretched, and extension (bending backwards) of the neck with chin tucked position (Fig 6).

Stretching:

Fig 2

Fig 3


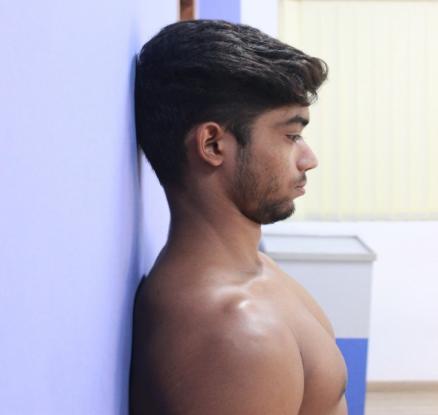

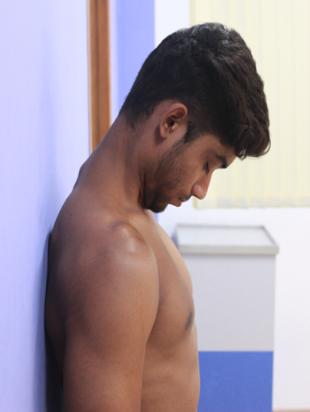


Cervical extensors

Sub-occipital muscles

Fig 5

Fig 6

Fig 4


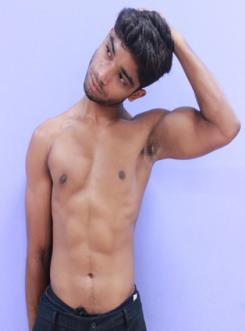

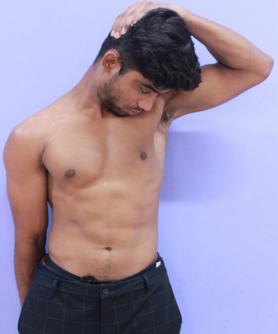

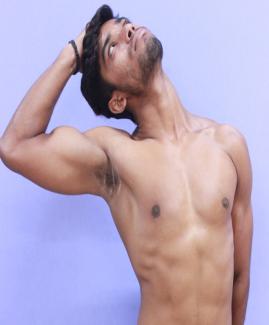


Sternocleidomastoid

Levator scapulae

Upper trapezius

*Re-education of the craniocervical flexion movement and recruitment of DNF:*

***Head nod:* (Cranio-cervical flexion)**

Standing against a wall and maintaining the contact, perform head nodding by placing the tongue on the roof of the mouth without activating the SCM and scalenes.

Instruction: While performing the head nod, slide the back of the head in cepalad direction (towards the ceiling) and to hold this position for 10 counts and repeated for 10 times, twice daily (Fig 7).

Fig 7


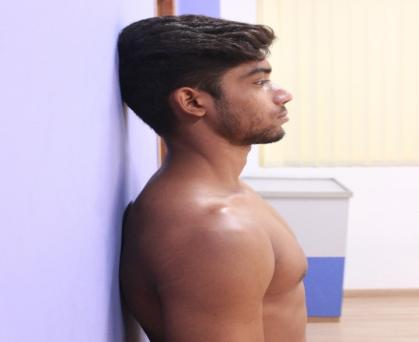

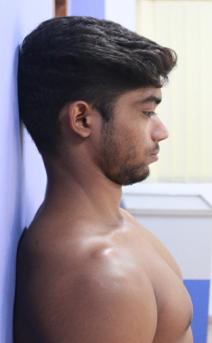


After re-educating the cranio-cervical movement, training the deep neck flexors will be done to improve the recruitment.

Segmental extension from slump position:

Beginning from a flexed slump position, segmental extension is performed in the following sequences


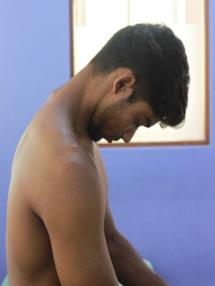

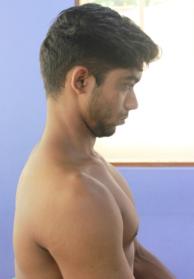

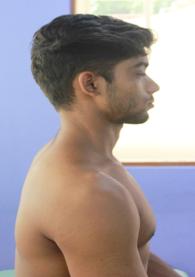


Upper cervical extension

Lower cervical extension

Starting with upper and lower cervical flexion

Total number of sets: 3-5 sets.

Progression of DNF activation:

Nod lift-off on incline surface Supine nod lift-off with Isometric hold Supine nod lift-off with isotonic contraction (Inner range cervical flexion) (Fernández-de-las-Peñas C & [Cleland JA](https://www.ncbi.nlm.nih.gov/pubmed/?term=Cleland%20JA%5BAuthor%5D&cauthor=true&cauthor_uid=18558274), 2011).


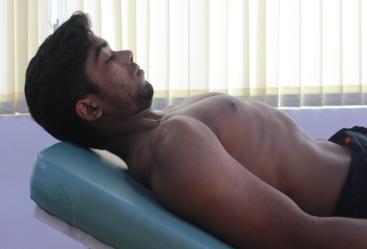

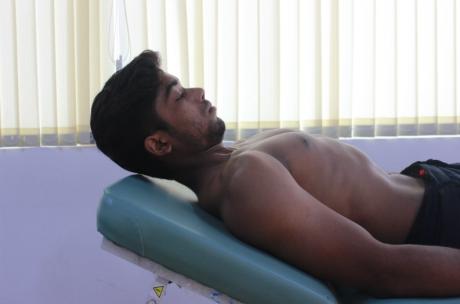


Nod lift-off on an incline surface with isometric hold isomer


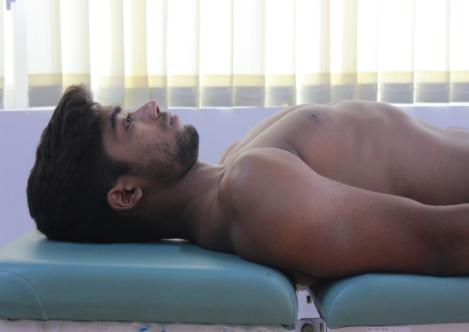

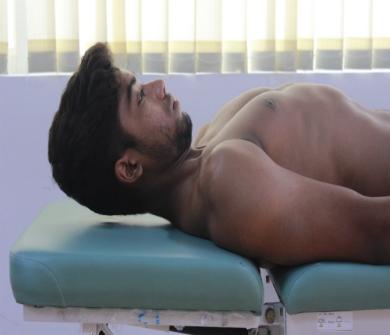


Nod lift-off in supine with isometric hold

Total number of sets: 3-5 sets

Isometric holding time: Initially begin with 3 seconds hold and then progress it to increasing the holding time.

*Progression of DNE activation:*

Segmental extension in quadruped


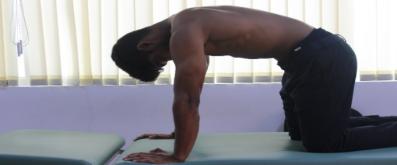

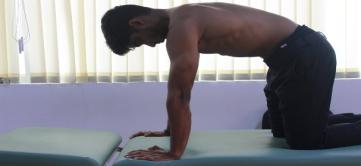

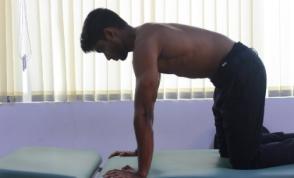


*Retraining scapular control:*

After retraining the scapular orientation, training to improve the endurance of the synergistic muscles of the scapula will be done in prone to achieve scapular control.

Progressive retraining of the scapular control can be done using different positions and movement of the arms in the position of T, V, W and L.

Total number of sets: 3-5 sets for each variations

Number of repetitions: 10 repetitions in each set.


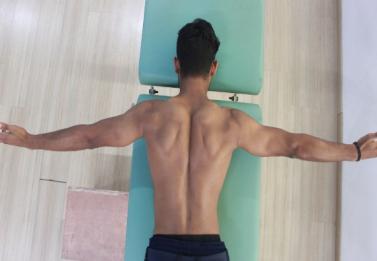

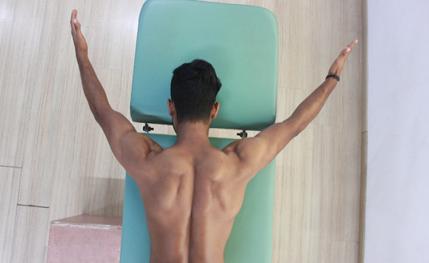


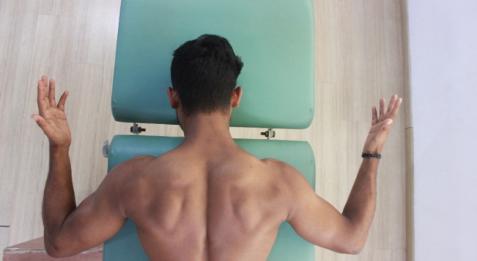

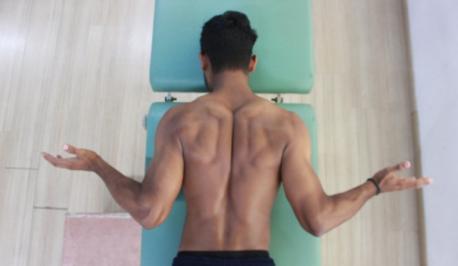


All these therapeutic exercises should be practiced atleast 5 days in a week from the initial treatment session till the end of 3^rd^ month.
