# Supplementary material for: Effectiveness of deep cervical fascial manipulation and yoga postures on pain, function, and oculomotor control in patients with mechanical neck pain: study protocol of a pragmatic, parallel-group, randomized, controlled trial
Source: Trials. 2021 Aug 28;22:574. doi: 10.1186/s13063-021-05533-w (PMC8399821; doi:10.1186/s13063-021-05533-w)
Supplement: Supplementary file 5 — Additional file 5. Yoga poses - Information leaflet. [file 13063_2021_5533_MOESM5_ESM.docx]

**Sequential yoga poses:**

The aim is to improve the mobility of the joints, flexibility of the muscles and the fascia of the neck and upper limb. Participants will be taught to perform different yoga poses in a sequence to target the myofascial lines.

It is OK to feel mild stretch pain during exercises as long as it is tolerable. If the symptoms flare up or persist for more than a day, reduce the intensity and correct the form of the poses. Resume the usual training routine, once the acute exacerbation of symptoms subsides.

For queries: contact Prabu Raja G, 8867591333, [prabu.raja@manipal.edu](mailto:prabu.raja@manipal.edu).

**
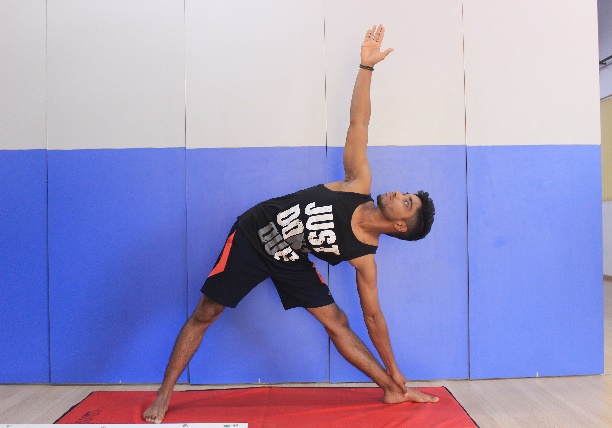

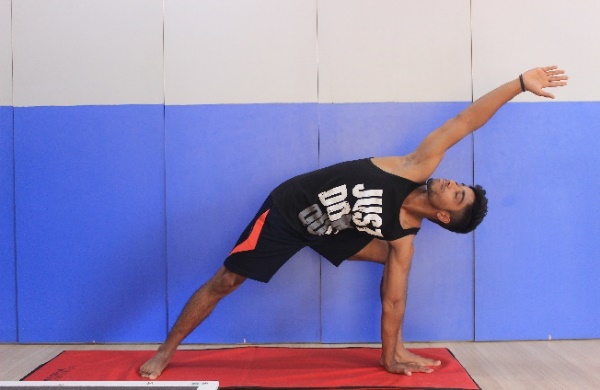
**

Pose-1 (Triangle Pose / Trikonasana) Pose-2 (Extended Side Angle Pose /

Utthita Parsvakonasana)

**
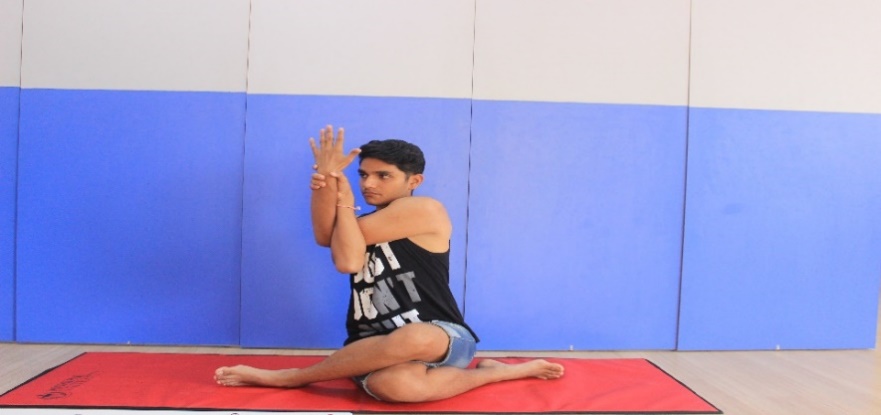

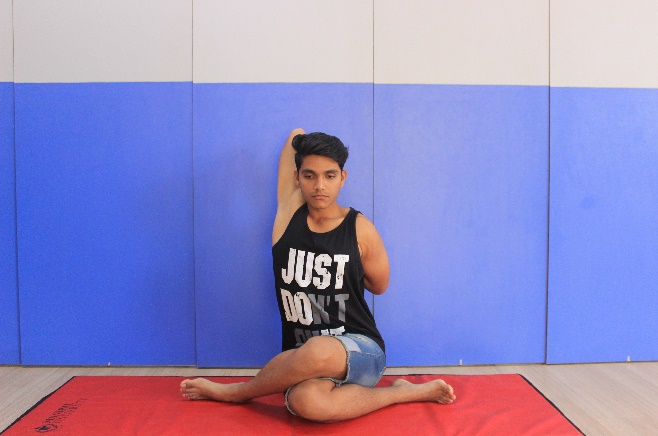
**

Pose-3 (Seated Eagle Pose / Garudasana) Pose-4 (Cow Face pose / Gomukhasana)

**
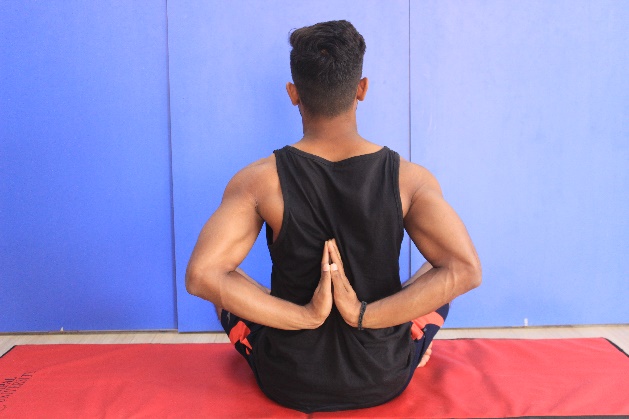

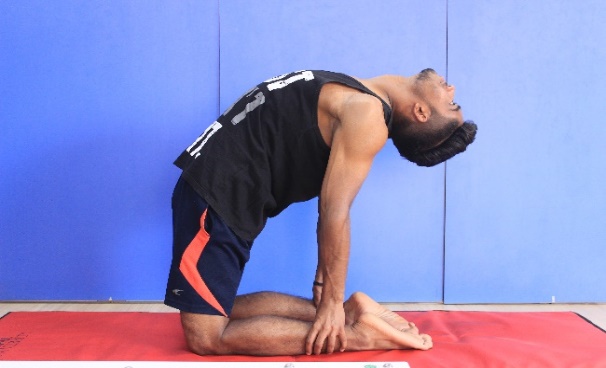
**

Pose -5 (Reverse Prayer Pose / Pose-6 (Camel Pose / Ustrasana)

Paschima Namaskarasana)

**
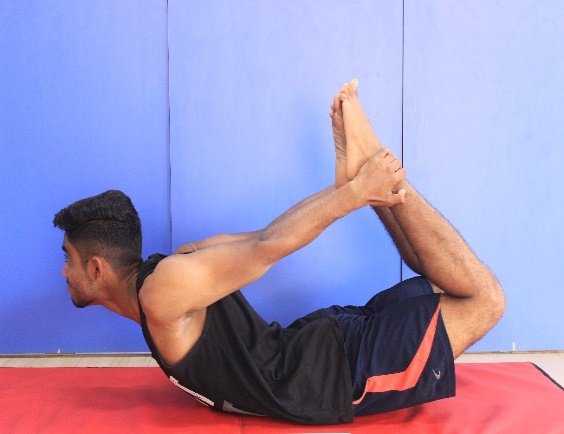

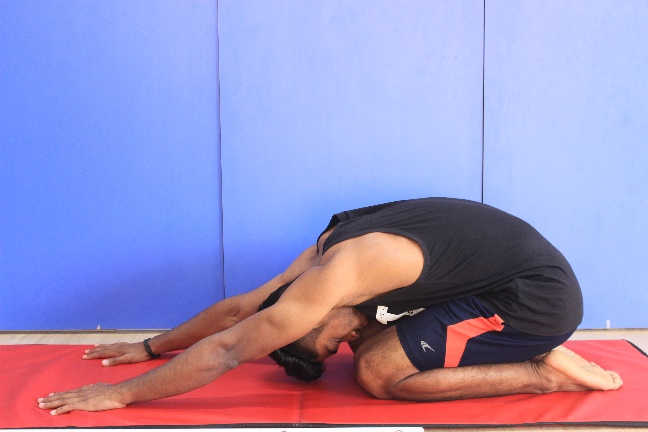
**

Pose-7 (Bow Pose / Dhanurasana) Pose-8 (Child Pose / Balasana)

Total number of sets: Two repetitions of each pose, on each sides beginning with the right side followed by left side. Each pose will be performed in the following order: Right 🡪 Left 🡪 Right 🡪Left

Yoga pose holding time: Initially begin with 5 breathes hold in each pose and then progress the holding time by increasing the number of breathes.
All these poses should be practiced atleast 5 days in a week, from the initial treatment session till the end of the 3^rd^ month before the final follow up evaluation..
